# A Sensitive LC-MS/MS Method for Palytoxin Using Lithium Cationization

**DOI:** 10.3390/toxins10120537

**Published:** 2018-12-14

**Authors:** Mirjam D. Klijnstra, Arjen Gerssen

**Affiliations:** RIKILT, Wageningen University and Research Center, Akkermaalsbos 2, 6708 WB Wageningen, The Netherlands

**Keywords:** palytoxin, ovatoxins, mass spectrometry, cationization

## Abstract

Palytoxin (PlTX) and analogues are produced by certain dinoflagellates, sea anemones, corals and cyanobacteria. PlTX can accumulate in the food chain and when consumed it may cause intoxication with symptoms like myalgia, weakness, fever, nausea, and vomiting. The analysis of PlTXs is challenging, and because of the large molecular structure, it is difficult to develop a sensitive and selective liquid chromatography-mass spectrometry (LC-MS/MS) method. In this work, an LC-MS/MS method was developed to analyse PlTXs with use of lithium iodine and formic acid as additives in the mobile phase. For method development, initially, LC-hrMS was used to accurately determine the elemental composition of the precursor and product ions. The main adduct formed was [M + H + 2Li]^3+^. Fragments were identified with LC-hrMS and these were incorporated in the LC-MS/MS method. A method of 10 min was developed and a solid phase extraction clean-up procedure was optimised for shellfish matrix. The determined limits of detection were respectively 8 and 22 µg PlTX kg^−1^ for mussel and oyster matrix. Oysters gave a low recovery of approximately 50% for PlTX during extraction. The method was successfully in-house validated, repeatability had a relative standard deviation less than 20% (*n* = 5) at 30 µg PlTX kg^−1^ in mussel, cockle, and ensis, and at 60 µg PlTX kg^−1^ in oyster.

## 1. Introduction

Palytoxin (PlTX), ovatoxin (OVTX), ostreocin, and other analogues are produced by the dinoflagellates *Ostreopsis ovata* and *Ostreopsis siamensis* [1,2,3,4] as well as by certain sea anemones, and corals of the *Palythoa* species and cyanobacteria of the genus *Trichodesmium* [5,6]. The *Ostreopsis* species is widely spread throughout the world [7]. From these producers, PlTXs can accumulate in the food chain in organisms like mussels, crabs, and fish [8]. PlTXs are one of the largest non-protein compounds within the class of marine biotoxins. These polyhydroxylated compounds with both lipophilic and hydrophilic parts consist out of a long, partially unsaturated, aliphatic backbone containing cyclic ethers, hydroxyl, and amide groups. Currently, more than 25 different PlTX analogues have been described such as the OVTX a-e, ostreocins and mascarenotoxins [9,10]. Since the discovery of PlTX in the 1960’s, a number of exposures and poisonings related to PlTX have been reported from several parts of the world [11,12]. Exposure to PlTXs was mostly related to inhalation exposure. The most well know intoxication event was in Italy in 2005 where over 200 people showed respiratory problems [13]. However, smaller incidents are also reported such as in the Netherlands where four people suffered from high fever and overall malaise after cleaning an aquarium, where stones were cooked in order to remove a colony of zoanthids [14]. PlTX binds to the extracellular part of the Na^+^/K^+^-ATPase, opening the cation channel, which inhibits the transport of Na^+^ and K^+^ across the cell membrane. This results in mitochondrial damage and cell death [15]. Furthermore, an increase in the cytosolic Ca^2+^, caused by the action of a Na^+^/Ca^2+^ exchange pump, is observed, which also eventually causes cell death [16]. Symptoms of PlTX intoxication are myalgia, weakness, fever, nausea, and vomiting. The toxicity of PlTX is strongly dependent on the route of administration; in animal experiments, the toxicity of PlTX is much lower after oral administration compared to intraperitoneal (i.p.) administration. Animal studies revealed an LD_50_ of <1 μg kg^−1^ via intravenous administration, however, it is approximately 1000-fold less toxic after oral administration (LD_50_ 767 µg kg^−1^) [17,18]. Currently, no international legislation has been established mentioning safe PlTX levels in shellfish. The European Food Safety Authority (EFSA) has reviewed the available toxicological data in 2009. They concluded based on the available data an acute reference dose (ARfD) of 0.2 µg kg^−1^ bodyweight. This ARfD can be translated to a limit of 30 µg PlTX-eq kg^−1^ in shellfish meat with the assumption of a person of 60 kg consuming 400 g of shellfish meat [19].

To detect PlTXs in shellfish, several methods have been described: In vivo methods, in vitro methods, immunological methods, and analytical chemical methods. The in vivo mousse bioassay (MBA) [20] is based on the i.p. injection of a shellfish extract. The observed effect is stretching of hind limbs and lower back, weakening of forelimbs, ataxia, decreased locomotion, convulsions, gasping for breath, and eventually death. In vitro assays are based on the toxicological effect on either a cell line or on receptor binding. The latter is based on the effect on the Na+/K+ ATPase pump which is labelled with a fluorescent label. This can be measured using fluorescence polarisation [21]. Other in vitro assays developed are for example based on a neuro2a cell line and the detection of cell viability based on 3-(4,5-dimethylthiazol-2-yl)-2,5-diphenyltetrazolium bromide (MTT) [22] or on haemolytic activity of palytoxin against erythrocytes [23]. Immunological methods are based on the recognition of a certain part of the PlTX molecule by an antibody, as for example described by Fraga et al. [24]. Each type of method has its advantages and disadvantages. The in vivo test has a direct response (mouse death) and proponents believe unknown toxins will be detected by this system. The drawback of this test, beside that the method is unethical, is the route of administration, which is i.p. instead of oral. This will result in discrepancies as adsorption and metabolism are not taken into account. Furthermore, the methodology has a poor selectivity towards which compound is responsible for the observed effect. The in vitro tests are a good alternative. A pro of the in vitro test is that no expensive standards solutions and animal facilities are required. However, an observed response will still need verification by a confirmatory method. Immunological methods are fast and sensitive. However, as only a part of the molecule is recognized by the antibody, cross reactivity towards PlTX analogues might be poor, causing an underestimation or an overestimation of the true PlTX concentration, and still confirmation of the toxin should be performed. For confirmatory purposes, analytical chemistry based on liquid chromatography coupled to mass spectrometry (MS) can be used. The drawback of analytical chemical methods is the need for individual toxin standards for each analogue [20]. Unfortunately, for PlTXs the developed chemical methods are not always sensitive enough to detect PlTXs at the EFSA recommended safe levels. Ciminiello et al. published a method based on liquid chromatography coupled to high resolution mass spectrometry (LC-hrMS) to obtain accurate masses in full scan mode. In the hrMS complex spectra were obtained consisting out of multiple adducts with various charge states and in source fragmentation of multiple losses of water. The sensitivity of this method is sufficient (limit of quantitation (LOQ) of 44 ng mL^−1^ in shellfish extract) but can be improved [25,26].

Selwood et al. developed a more sensitive method based on a micro oxidation of PlTX to low molecular weight oxidation products [27]. These oxidation products are then analysed by LC-MS/MS. The drawback of this method is that various PlTX analogues will give the same oxidation products which hamper selectivity. This selectivity is important as not all PlTX analogues will have the same toxicological potency [28]. Therefore, in this paper a sensitive method based on the ionization of intact PlTX is presented. The principle is based on cationization, this can be accomplished by the addition of alkali-metal ions such as lithium to the mobile phase. The alkali-metal ions will form a chelate or complex with the oxygen groups in the molecule. This approach has been described for somewhat larger molecules in various application fields such as the analysis of polysaccharides, polymers, and oils [29,30,31].

## 2. Results

### 2.1. Infusion Experiments with High Resolution Mass Spectrometry

**Figure 1:**
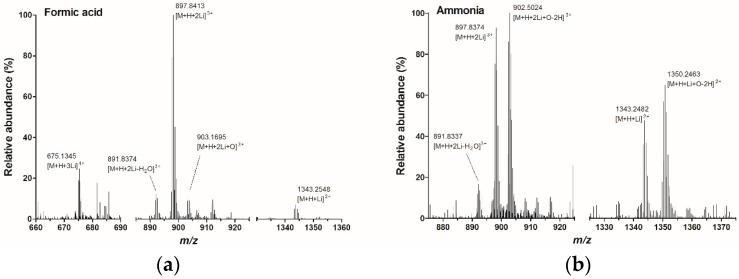
Ionisation of PlTX with LiI under (**a**) acidic and (**b**) alkaline conditions, masses shown are the monoisotopic *m/z* values.

In order to obtain the optimal conditions for the cationization, infusion experiments were carried out with different mobile phase compositions. The LC flow of 0.1 mL min^−1^ and a concentration of 1 µg mL^−1^ PlTX were kept constant during all infusion experiments. The initial infusion experiments with hrMS were performed with a mobile phase containing formic acid and ammonium formate. Spectra in positive and negative ionisation mode contained, respectively, [M + H + Na]^2+^ and [M − 2H]^2−^ ions with relatively low abundance (spectra in Supplementary Material Figures S1 and S2). For cationization, LiI or LiCl were added to the mobile phase. Ionization efficiency under different pH conditions were investigated by adding various additives to the mobile phase. For acidic conditions formic acid was added, for alkaline conditions ammonia was added and also the mobile phase without additive was tested. The concentration of the cationizing agent varied from 0.1 to 2 mM, and the concentration of the additive varied from 0.0005% to 0.01%. During the initial experiment, a full MS scan of *m/z* 700 to 2800 was acquired in both positive and negative electrospray ionisation (ESI) mode. With ESI negative mode no PlTX ions were formed when the mobile phase contained Li. With positive ESI mode, PlTX ions were formed without additives, and under both acidic and alkaline conditions. However, no single charged ion species was obtained (Figures S3–S8). Therefore, in all further full scan MS experiments, spectra were acquired with an *m/z* 500 to 1400 in positive ESI mode. Figure 1 shows all major ions formed with formic acid or ammonia as an additive. The ions formed without additives in the mobile phase were similar to the ions under acidic conditions, only lower in abundance. Despite the fact that due to the high number of carbon elements, C_129_H_223_N_3_O_54_, the ^13^C isotopic mass of PlTX is higher in abundance compared to the monoisotopic mass, all *m/z* values mentioned in the article and in the figures are based on the monoisotopic masses in order to avoid confusion about elemental compositions.

Under acidic conditions one major ion was formed: *m/z* 897.8413, which corresponds to the [M + H + 2Li]^3+^ ion. Furthermore, *m/z* 903.1695 ([M + H + 2Li + O]^3+^), *m/z* 891.8374 ([M + H + 2Li − H_2_O]^3+^), *m/z* 675.1345 ([M + H + 3Li]^4+^), and *m/z* 1343.2548 ([M + H + Li]^2+^) were observed. Under alkaline conditions two major ions were formed: *m/z* 897.8374 ([M + H + 2Li]^3+^) and *m/z* 902.5024 ([M + H + 2Li − 2H + O]^3+^). Furthermore, low abundant ions with *m/z* 1343.2482 ([M + H + Li]^2+^) and *m/z* 1350.2463 ([M + H + Li − 2H + O]^2+^) as well as some in-source fragmentation resulting in multiple losses of water were detected. No quadruple charged ions were formed under alkaline conditions. The average Δ ppm error of the measured masses compared to the theoretical masses was 2.5 ppm with a maximum of 4.1 ppm for the [M + H + Li − 2H + O]^2+^ ion and a minimum of 1.6 ppm for the [M + H + 2Li + O]^3+^ ion. Under both the acidic and alkaline conditions, an in-source oxidation product was observed, the [M + H + 2Li + O]^2+^ and [M + H + Li − 2H + O]^2+^ ion under respectively acidic and alkaline conditions. This in-source oxidation which occurred is not very often described, however, there is some literature available on the oxidation of large molecules. A possible cause might be the condition of the ESI capillary, which is described by Chen et al. who showed that corroded capillaries can cause in-source oxidation of peptides [32]. The effect of the counter ion was studied by applying two different Li salts, LiI and LiCl. Both salts gave the same PlTX ions in the spectra. However, when LiI was used in the mobile phase there was a higher abundance in the PlTX ions of interest. Finally, the formic acid concentration in the mobile phase was optimized. The highest abundance of the [M + H + 2Li]^3+^ ion was found with 0.25 mM LiI in combination with 0.00125% formic acid.

### 2.2. Fragmentation PlTX with High Resolution Mass Spectrometry

**Figure 2:**
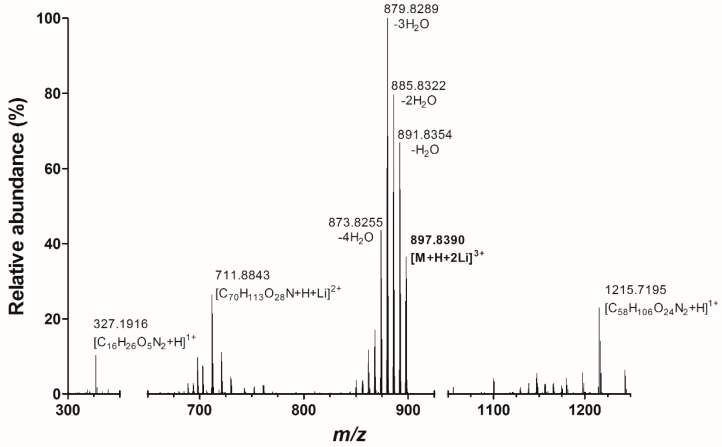
Fragmentation spectrum of PlTX under acidic conditions with LiI in electrospray ionisation (ESI) positive mode.

**Figure 3:**
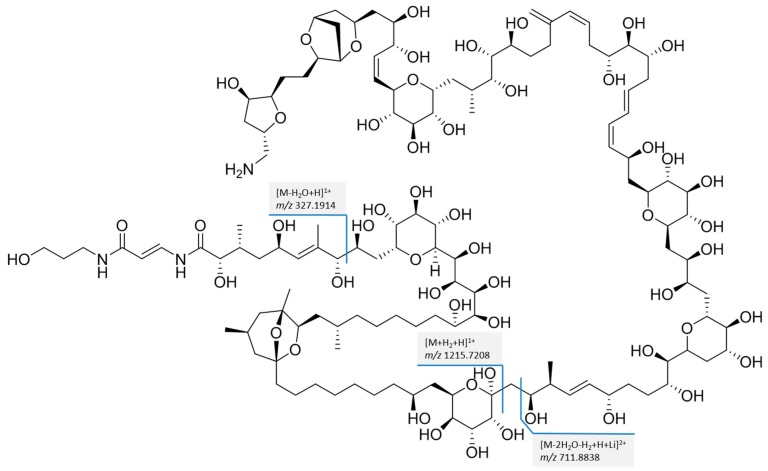
Proposed fragmentation of PlTX under acidic conditions with LiI.

When selecting *m/z* 898 as the precursor ion, the fragmentation spectrum as shown in Figure 2 was obtained. A series of water losses were observed with *m/z* 891.8354 (−H_2_O), *m/z* 885.8322 (−2H_2_O), *m/z* 879.8289 (−3H_2_O), and *m/z* 873.8255 (−4H_2_O). Furthermore, more structural specific product ions with *m/z* 1215.7195, *m/z* 711.8843, and *m/z* 327.1916 were obtained. Elemental compositions of these ions are respectively [C_58_H_106_O_24_N_2_+H]^1+^, [C_70_H_113_O_28_N+H+Li]^2+^ and [C_16_H_26_O_5_N_2_+H]^1+^. The proposed structures of the fragments are shown in Figure 3.

### 2.3. Chromatography

**Figure 4:**
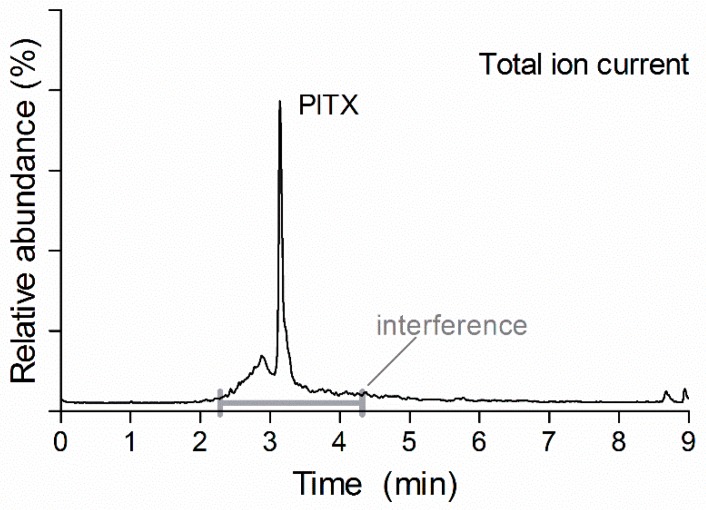
Chromatography PlTX 100 µg kg^−1^ in mussel with 10 min screening method.

**Figure 5:**
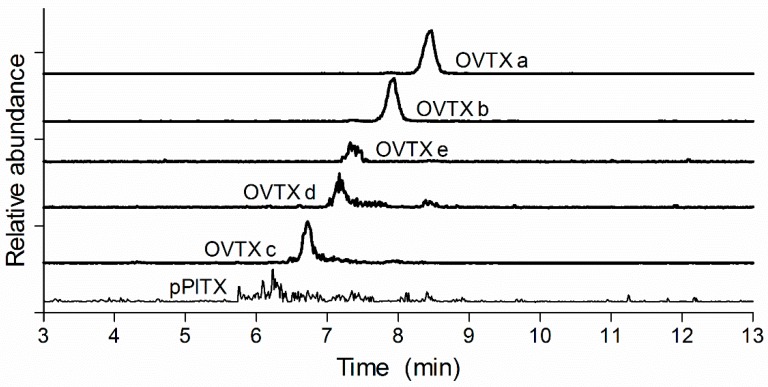
Separation of ovatoxins (OVTXs) a, b, c, d, e and putative PITX (pPlTX) in algae extract.

To rapidly screen for PlTXs a 10 min LC method was developed, as shown in Figure 4, where the total ion current (TIC) is shown. Although most PlTXs are separated by time or *m/z*, some of the OVTXs have the same *m/z* and are structurally similar and are therefore not separated with the fast 10 min LC gradient. To obtain an improved chromatographic separation a slightly longer LC-method with a gradient with a slower increase over time in organic strength was developed. The longer LC method is capable of separating the OVTXs a to e and putative PlTX (pPlTX) as demonstrated for an algae extract (Figure 5).

### 2.4. Sample Clean-Up

**Figure 6:**
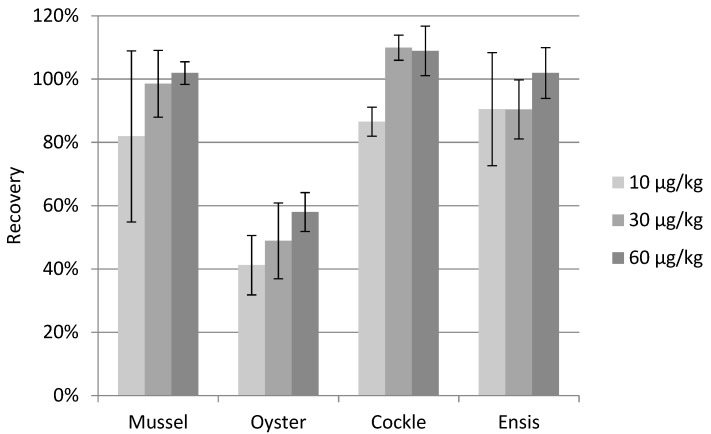
Recovery and relative standard deviation (RSD) of PlTX in shellfish matrix (*n* = 5).

For method development of the extraction and clean-up procedure, blank mussel and oyster homogenates were fortified with 60 µg PlTX kg^−1^. During method development, oyster gave a low recovery varying between approximately 5 and 50%. By performing spike experiments at different stages in the procedure it could be concluded that the low recovery of PlTX in oyster occurred during the extraction and not during the solid phase extraction (SPE) clean-up procedure. Besides the methanol/water (50:50 *v*/*v*) extraction, a variety of extraction solvents was tried like 100% methanol or methanol/water (50:50 *v*/*v*) with additives at various pHs. Furthermore, the addition of ethylenediaminetetraacetic acid (EDTA) was explored to improve extraction efficiency. EDTA was added to create metal complexes and reduce the amount of free metals like zinc in the extract which might also form complexes with PlTX. This might be the case as it is well known that oysters are rich in zinc content compared to other shellfish species [33]. Unfortunately, recoveries did not improve with the tested procedures, therefore it was decided to continue with the methanol/water (50:50 *v*/*v*) extraction solvent. The low observed recoveries of PlTX in oyster were also obtained during the validation study (Figure 6). When extracting with methanol/water (50:50 *v*/*v*) and performing SPE clean-up the limit of detection (LOD) for PlTX in mussel and oyster are respectively 8 and 22 µg kg^−1^. 

### 2.5. Method Validation

In order to validate the developed quantitative LC-MS/MS method twenty-one samples were used to determine selectivity, linearity, recovery, and repeatability. Validation was performed according to the SANTE/2015/11945 guidance document [34]. Solely PlTX was validated as no other commercial standards for the analogues and OVTXs are available.

The selectivity of the method was determined by the analysis of 20 blank shellfish samples (five mussels, five oysters, five ensis and five cockles). According to the SANTE document, the interferences present should be less than 30% of the PlTX peak area. During the validation, the water loss multiple reaction monitoring (MRM) transitions used, 898.2 > 880.2 and 898.2 > 874.2, showed background interference in both mussel and oyster (Figure 4). The area of the background in the samples spiked with 10 µg PlTX kg^−1^ exceeded the 30%, at higher concentrations, >30 µg PlTX kg^−1^, this improved. With the other MRM transition used, 897.8 > 711.9, less background interference was obtained. However, LODs with the water loss MRMs were much lower compared to the other MRM transitions and therefore the water loss MRMs were used for quantification. Furthermore, it can be argued that these water losses were not specific. From a triply charged ion, a single loss of water is a loss of *m/z* 6, which is not possible to observe from single and doubly charged molecules. Although, the loss of 3H_2_O, MRM transition 898.2 > 880.2, is the loss of *m/z* 18, which is the same as the loss of one H_2_O in a single charged molecule. This might explain the relatively high interference background for this transition. However, the loss of 4H_2_O corresponds to a *m/z* 24 loss which is not a commonly obtained neutral loss. For additional confirmation, a third transition (897.8 > 711.9) was used, however, this transition was not used for quantitation. Both ensis and cockle showed the same kind of background signal as mussel and oyster. However, it was less in abundance and therefore when spiked with 10 µg PlTX kg^−1^ the area of the interference was less than 30% of the PlTX area.

Linearity was determined by constructing a calibration curve with five matrix matched standards ranging from 5 to 100 µg PlTX kg^−1^ in mussel homogenate. The requirement for the correlation coefficient was >0.990. During the validation, the correlation coefficient was ≥0.997 and therefore it met the requirements. Furthermore, as a requirement, the residuals from the regression line should be below 20%. Only the 5 µg PlTX kg^−1^ standard did not meet this criterion as a residual of −27% was observed. This is also in line with the LOQ of 8 µg PlTX kg^−1^ in mussel caused by the relatively high background signal.

To calculate the recovery, all various shellfish matrices were quantified using the calibration curve constructed using mussel matrix matched standards. The recovery of mussels, cockles and ensis were within the requirements of >70% and <120% (Figure 6). The recovery of PlTX in oysters was on average 50%. For the repeatability error, the relative standard deviation (RSD) should be below 20%. The RSD of the repeatability exceeded the 20% in mussel fortified at 10 µg PlTX kg^−1^ and in oysters at 10 and 30 µg PlTX kg^−1^ due to the background signal and relative low recovery in oysters.

## 3. Discussion

The analysis of PlTXs is challenging, because of the large molecular structure it is difficult to develop a sensitive and selective LC-MS method. A method was successfully developed to analyse PlTXs in shellfish with LC-MS/MS. Improved sensitivity was obtained using cationization with lithium. By application of this, the LODs were respectively 8 and 22 µg PlTX kg^−1^ for mussels and oysters. The method was successfully in-house validated at levels of 30 and 60 µg PlTX kg^−1^. Currently, no legislation is established for PlTXs, however, based on the EFSA opinion, safe consumption levels should be below 30 µg PlTX-eq kg^−1^ of edible shellfish. Therefore, the developed method can be used for the analysis on the levels as mentioned in the EFSA opinion.

Compared to previous described LC-MS methods, i.e., Ciminiello et al. and Selwood et al., sensitivity is slightly improved or comparable [25,27]. The hrMS method by Ciminiello et al. provided more complex spectra compared to our more simplified spectra which resulted in improved LODs. Although the method of Selwood et al. is comparable in sensitivity with an LOQ of 10 µg PlTX kg^−1^ in seafood, the selectivity is greatly improved. With the developed method intact, PlTX molecules and analogues can be analysed compared to the generic oxidation products as done by Selwood et al. Our method will give more insight into the toxin profile, which might be important as not all analogues pose the same toxicological potency.

Furthermore, another marine biotoxin class was also tested with the same method. These are the ciguatoxins (CTXs). Current LC-MS/MS methodologies for CTXs would benefit from a gain in sensitivity, as the main challenge with the analysis of CTXs is the low LOQs required. This is because the recommended safe concentrations by the US food and drug administration as well as the EFSA are 0.01 μg equivalents of Pacific-CTX-1 kg^−1^ of fish. CTXs analysed with straightforward LC-MS/MS methods gave relatively poor ionization and fragmentation due to the structure of CTXs. Although recently Moreiras et al. [35] published a systematic approach to optimize ionisation for CTXs, which already slightly improved sensitivity, an attempt was made with the formation of possible lithium adducts. Unfortunately, no detectable lithium ions were formed when CTX standards were analysed with this method, most likely due to the limited availability of hydroxyl groups in the CTX structure.

## 4. Materials and Methods

### 4.1. Chemicals and Materials

Formic acid (98–100%), acetic acid (96%), and ammonia (25%) were purchased from VWR, Amsterdam, the Netherlands. Acetonitrile (Ultra LC-MS), methanol (Ultra LC-MS), and water (Ultra LC-MS) were purchased from Actu-All, Oss, the Netherlands. Lithium iodide hydrate (98%) and lithium chloride (>99%) were purchased from Sigma-Aldrich, Zwijndrecht, the Netherlands. PlTX (>90%) was purchased from Wako, Osaka, Japan.

Blank shellfish samples used for method validation were collected in 2017 from several shellfish production areas in the Netherlands. Contaminated sample extracts containing pPlTX and OVTXs were a gift from Carmela Dell’aversano, University of Naples Federico II, Napoli, Italy.

### 4.2. Preparation of Standards

The PlTX standard was dissolved to a concentration of 20 µg mL^−1^ in methanol/water (50:50 *v*/*v*). Before the spiking experiments, the stock solution was further diluted to 1 µg mL^−1^ with methanol/water (50:50 *v*/*v*).

### 4.3. Liquid Chromatography Coupled with Mass Spectrometry

#### 4.3.1. Sample Clean-up

1.0 ± 0.05 g shellfish tissue homogenate was weighed and extracted with 3 mL methanol/water (50:50 *v*/*v*). The sample was vortex mixed for one minute using a multi-pulse vortex. The sample was centrifuged at 2,000× *g* for 5 min and the supernatant was decanted from the pellet to a new tube. The extraction procedure was repeated twice and supernatants were combined.

A Strata-X polymeric reversed phase cartridge of 60 mg (Phenomenex, Utrecht, the Netherlands) was conditioned and equilibrated with 3 mL methanol followed by 3 mL water. The sample extract of 9 mL was applied to the cartridge and subsequently washed with 3 mL water. PlTXs were eluted with 3 mL methanol containing 0.1% acetic acid. The eluent was transferred into a glass vial and used for analysis with LC-MS.

#### 4.3.2. Liquid Chromatography Methods

Chromatographic separation of PlTXs was achieved on a Kinetex C_18_ 1.7 µm, 2.1 x 100 mm column (Phenomenex, Utrecht, the Netherlands). Mobile phase A consisted of water, and mobile phase B consisted of acetonitrile/water (9:1 *v*/*v*), both containing 0.25 mM lithium iodide and 0.00125% formic acid. The injection volume was set at 10 µL. The column temperature was set at 40 °C and the total run time was 10 min. The gradient elution with a flow of 0.4 mL min^−1^ was as follows: 0.5 min at 10% mobile phase B, then linearly increased to 100% mobile phase B in 5.5 min and kept at 100% mobile phase B for 1.9 min. Subsequently, the gradient went back to 10% mobile phase B in 0.1 min and kept at 10% mobile phase B for 2 min to equilibrate the column for the next run.

An additional 15 min LC-gradient was developed with the same column, mobile phases, and settings to separate OVTXs when necessary. The flow was kept for 0.5 min at 100% mobile phase A, then mobile phase B was linearly increased to 28% in 0.5 min. In the next 12 min, mobile phase B was linearly increased to 32%. Subsequently, mobile phase B was linearly increased to 100% in 0.5 min and was kept at 100% mobile phase B for 0.5 min. The gradient went back to 100% mobile phase A in 0.1 min and kept at 100% mobile phase A for 0.9 min to equilibrate the column for the next run.

#### 4.3.3. High Resolution Mass Spectrometry

For method development, a Thermo Scientific UltiMate 3000 LC-system coupled to a Thermo Scientific Q Exactive focus hybrid quadrupole-orbitrap mass spectrometer (Thermo Fisher Scientific, Waltham, MA, USA) was used. In order to detect the PlTXs, electrospray ionisation in positive was used. The spray voltage in positive ionisation mode was set at 3.5 kV. The capillary temperature was set at 260 °C. For infusion experiments, PlTX was infused directly into the Mass spectrometer together with a flow of 0.1 mL min^−1^ mobile phase containing 50% acetonitrile and additives. A full MS scan event of 500 to 1400 *m/z* with a resolution of 70,000 full width at half maximum (FWHM) was acquired. In order to obtain fragmentation spectra of PlTX, fragmentation spectra were also acquired. The so called MS^2^ scans were obtained by selecting mass 898 ([M + H + 2Li]^3+^) with an isolation window of 4 Da. As collision gas nitrogen was used. The normalized collision energy (NCE) was set at 20 during fragmentation. Then after fragmentation, the ions were scanned from 187 to 1850 *m/z* with a resolution set at 70,000 FWHM. The automatic gain control representing the maximum capacity of the C-trap was set at a maximum of 10^6^ ions or a maximum injection time of 200 ms for the full scan and a maximum of 2 × 10^5^ ions or a maximum injection time of 200 ms for the MS^2^ scans were allowed.

#### 4.3.4. Triple Quad Mass Spectrometry

For measurements of shellfish extracts and the validation study, a Waters Acquity UPLC coupled to a Waters Xevo TQ-S tandem mass spectrometer (Waters, Milford, MA, USA) was used. The triple quad MS system was operated in positive electrospray mode and data were recorded in MRM mode using three transitions per toxin. PlTX: 898.2 > 880.2 collision energy (CE) 25 eV, 898.2 > 874.2 CE 25 eV, 897.8 > 711.9 CE 30 eV; OVTX a: 887.6 > 869.6 CE 25 eV, 887.6 > 863.6 CE 25 eV, 887.2 > 327.2 CE 30 eV; OVTX b: 902.2 > 884.2 CE 25 eV, 902.2 > 878.2 CE 25 eV, 901.9 > 371.2 CE 30 eV; OVTX c: 907.6 > 889.6 CE 25 eV, 907.6 > 883.6 CE 25 eV, 907.2 > 703.9 CE 30 eV; OVTX d and e: 892.8 > 874.8 CE 25 eV, 892.8 > 868.8 CE 25 eV, 892.5 > 327.2 CE 30 eV (specific for OVTX d) and 892.5 > 343.2 CE 30 eV (specific for OVTX e). Furthermore, a cone voltage of 30 V, a capillary voltage of 3 kV, source temperature of 150 °C, desolvation temperature of 600 °C and desolvation gas flow of 800 L h^−1^ was set.

### 4.4. Method Validation

Validation of the LC-MS/MS method for quantification of PlTX in shellfish was done according to the SANTE/2015/11945 guidance document [34]. There were twenty samples including mussel (*Mytilus edulis*), five oysters (*Castostrea gigas* now *Magallana gigas*), five ensis (*Ensis sp.*) and five cockles (*Cerastoderma edula*) homogenates were spiked each with 10, 30 and 60 µg PlTX kg^−1^ for determination of recovery and repeatability. The same 20 blank samples were used to determine selectivity. Matrix matched standards in mussel homogenate at 0, 5, 10, 30, 60 and 100 µg PlTX kg^−1^ were used for quantification and linearity.

## Supplementary Materials

The following are available online at http://www.mdpi.com/2072-6651/10/12/537/s1. Figure S1. Spectrum palytoxin (PlTX), electrospray ionisation (ESI) positive, mobile phase: Acetonitrile, H_2_O, formic acid, ammonium formate. Figure S2. Spectrum PlTX, ESI negative, mobile phase: Acetonitrile, H_2_O, formic acid, ammonium formate. Figure S3. Spectrum PlTX, ESI positive, mobile phase: Acetonitrile, H_2_O, LiI, formic acid. Figure S4. Spectrum PlTX, ESI negative, mobile phase: Acetonitrile, H_2_O, LiI, formic acid. Figure S5. Spectrum PlTX, ESI positive, mobile phase: Acetonitrile, H_2_O, LiI. Figure S6. Spectrum PlTX, ESI negative, mobile phase: Acetonitrile, H_2_O, LiI. Figure S7. Spectrum PlTX, ESI positive, mobile phase: Acetonitrile, H_2_O, LiI, ammonia. Figure S8. Spectrum PlTX, ESI negative, mobile phase: Acetonitrile, H_2_O, LiI, ammonia.
