# Supplementary material for: A Sensitive LC-MS/MS Method for Palytoxin Using Lithium Cationization
Source: Toxins (Basel). 2018 Dec 14;10(12):537. doi: 10.3390/toxins10120537 (PMC6316396; doi:10.3390/toxins10120537)
Supplement: Supplementary file 1 [file toxins-10-00537-s001.pdf]

Supplementary Materials: A Sensitive LC-MS/MS Method for Palytoxin Using Lithium Cationization

Mirjam D. Klijnstra and Arjen Gerssen

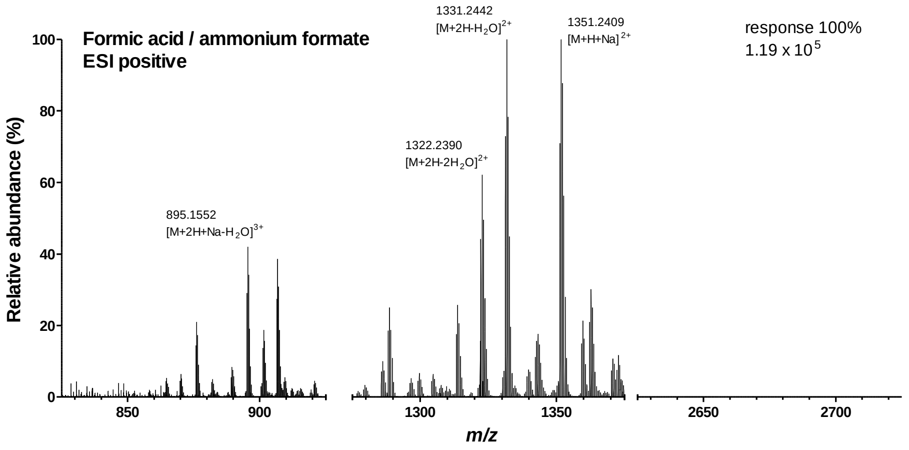

Figure S1. Spectrum palytoxin (PlTX), electrospray ionisation (ESI) positive, mobile phase: Acetonitrile, H_2O, formic acid, ammonium formate.

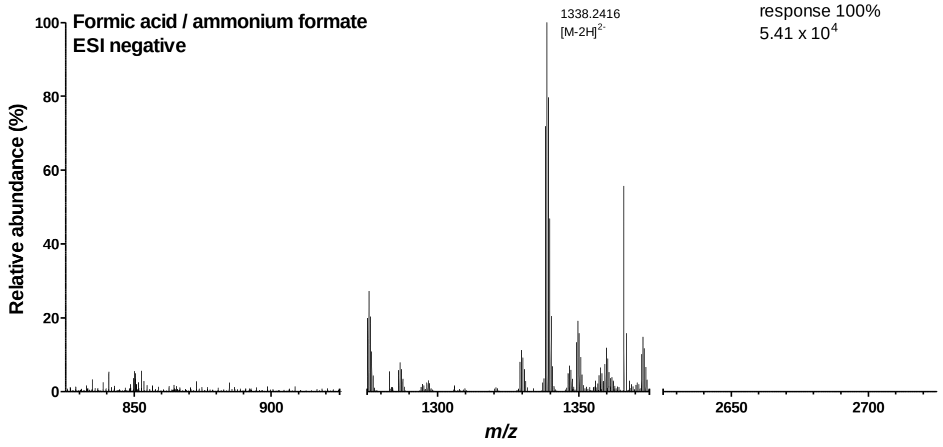

Figure S2. Spectrum PlTX, ESI negative, mobile phase: Acetonitrile, H_2O, formic acid, ammonium formate.

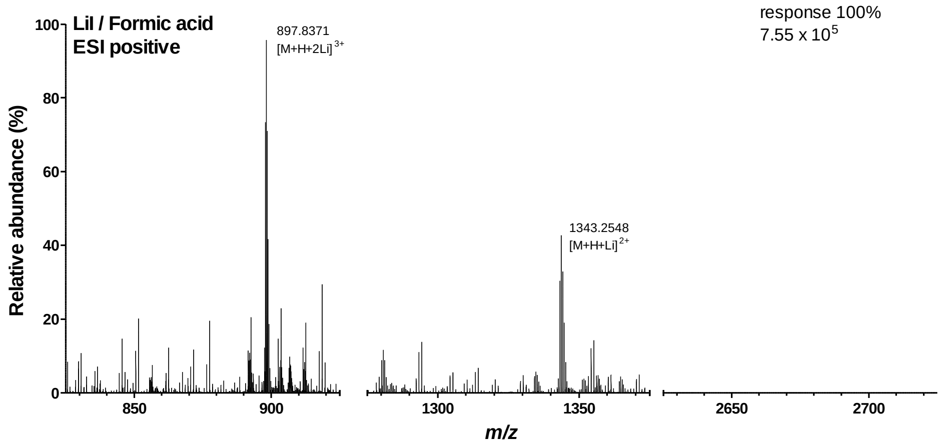

Figure S3. Spectrum PlTX, ESI positive, mobile phase: Acetonitrile, H_2O, LiI, formic acid.

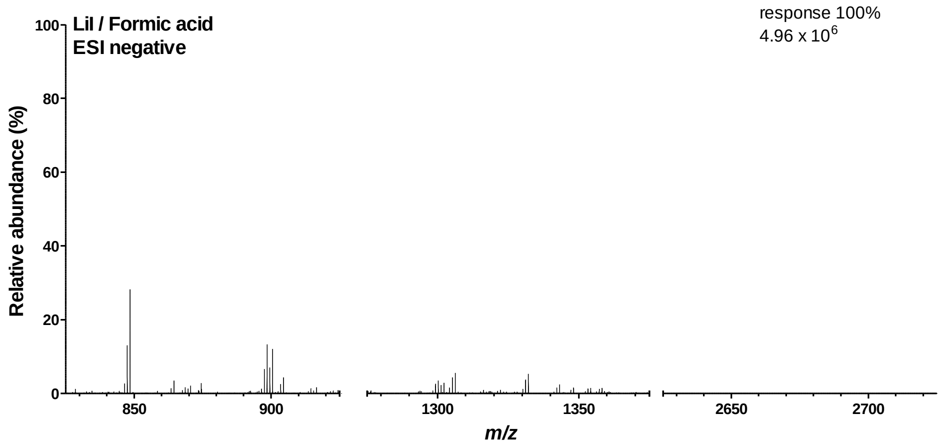

Figure S4. Spectrum PlTX, ESI negative, mobile phase: Acetonitrile, H_2O, LiI, formic acid.

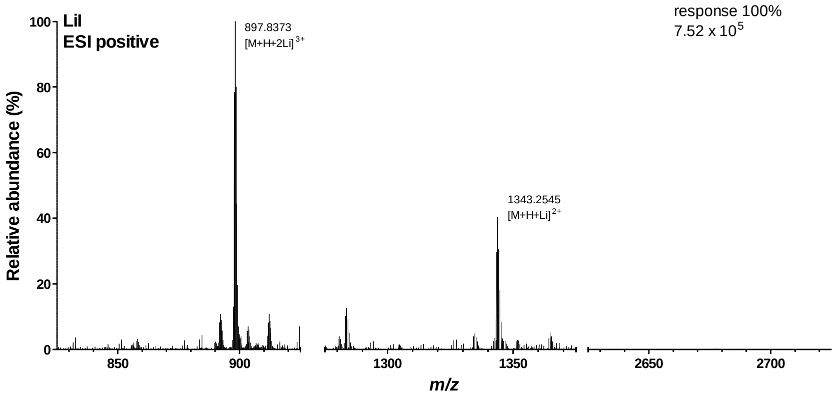

Figure S5. Spectrum PlTX, ESI positive, mobile phase: Acetonitrile, H_2O, LiI.

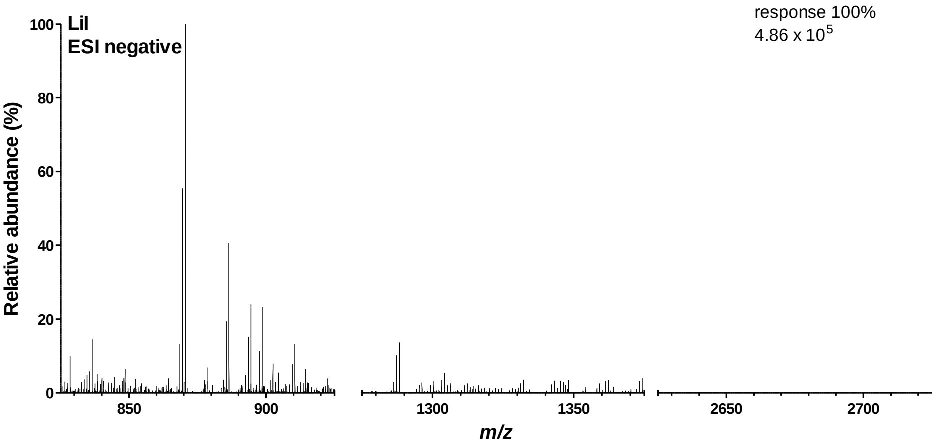

Figure S6. Spectrum PlTX, ESI negative, mobile phase: Acetonitrile, H_2O, LiI.

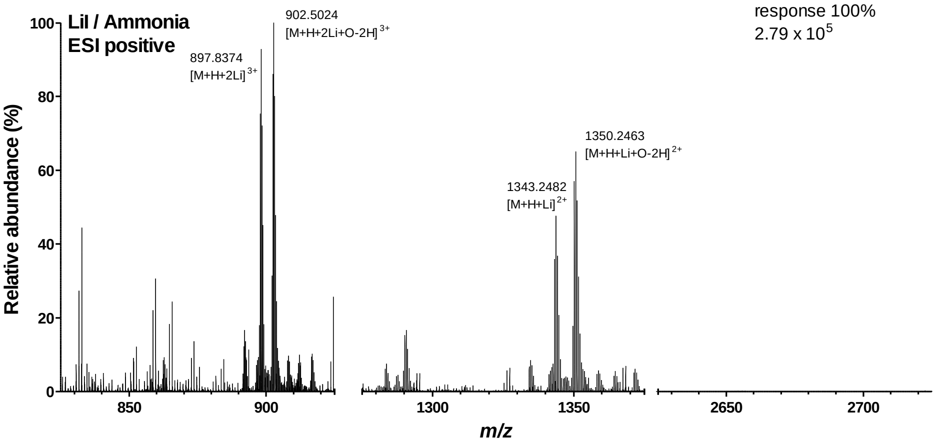

Figure S7. Spectrum PlTX, ESI positive, mobile phase: Acetonitrile, H_2O, LiI, ammonia.

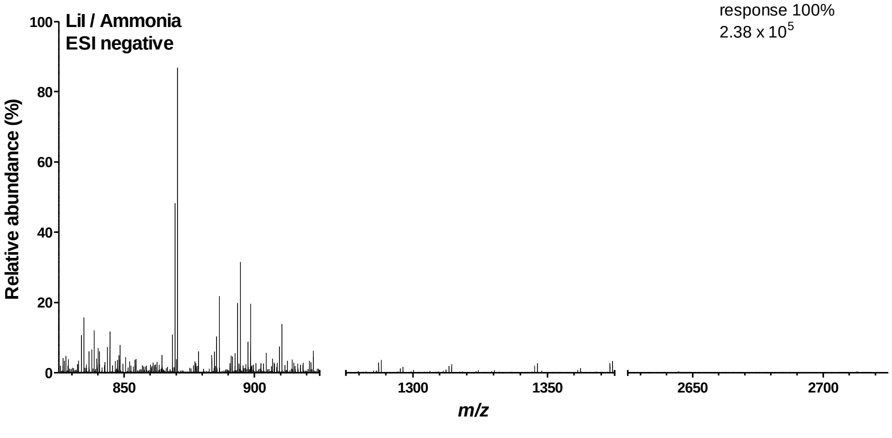

Figure S8. Spectrum PlTX, ESI negative, mobile phase: Acetonitrile, H_2O, LiI, ammonia.
